# Digital transformation and corporate ESG performance: Research based on a capability-motivation dual framework

**DOI:** 10.1371/journal.pone.0325295

**Published:** 2025-06-11

**Authors:** Cuicui Huang

**Affiliations:** 1 Department of Global Convergence, Kangwon National University, Chuncheon-si, South Korea; South China Normal University School of Economics and Management, CHINA

## Abstract

This study systematically examines the impact mechanism and heterogeneous characteristics of digital transformation (DT) on corporate environmental, social, and governance (ESG) performance, using panel data from Chinese A-share listed companies from 2010–2022. The research constructs a “capability-motivation” analytical framework based on resource dependence theory and agency theory, yielding the following conclusions. First, DT has a significant positive effect on corporate ESG performance, which is robustly verified through instrumental variables method and system GMM, among others. Second, DT enhances corporate ESG performance through the dual pathways of alleviating financing constraints (resource effect) and reducing agency costs (governance effect). Third, this promotional effect varies significantly across different lifecycle stages, being more prominent in mature and declining companies but not significant in growth-stage companies. Finally, the positive impact of DT on ESG performance is stronger in state-owned enterprises than in non-state-owned enterprises. This study integrates scattered findings in existing literature through a “capability-motivation” dual framework, providing a more systematic explanation for understanding the relationship between DT and ESG, and offering theoretical foundations and practical implications for companies to formulate differentiated digital-ESG strategies.

## 1. Introduction

In the strategic transformation period of high-quality economic development, ecological environmental protection has transformed from the traditional perception of a “cost burden” to a core engine driving sustainable growth. As the backbone of the capital market, listed companies are not only important contributors to economic prosperity but also pioneers in leading social green transformation [1]. Under the global sustainable development wave, corporate environmental, social, and governance (ESG) performance has become a key dimension for investors, regulatory agencies, and the public to evaluate corporate value [2–4]. Empirical research shows that excellent ESG performance can not only enhance corporate value [5,6] but also optimize financing structure [7] and promote corporate innovation [8,9]. Against the backdrop of building a modern economic system and implementing new development concepts, listed entities can achieve a win-win situation for both their business value and social contribution by improving ESG strategies and enhancing sustainable management capabilities, which will also provide strong momentum for driving the national economy toward a high-quality development track.

In recent years, academic exploration of the association between digital transformation (DT) and corporate ESG performance has shown a flourishing development trend, but research conclusions contain significant contradictions. The mainstream view holds that the two have a linear positive correlation, with DT promoting ESG performance improvement by enhancing green innovation capabilities, increasing information transparency, and optimizing corporate governance structures [10–14]. However, Yang and Han [15] propose a challenging perspective, finding that this relationship is actually an inverted U-shape—moderate DT benefits ESG performance, while excessive transformation produces negative effects due to organizational conflicts and increased costs. Furthermore, this correlation is also moderated by internal characteristics such as enterprise ownership nature, industry attributes [11,14], development stage [13], governance structure [11], as well as external environmental factors including government policy support [11], environmental uncertainty [10], and market development level [11]. Scholars have reached inconsistent conclusions regarding the relationship between DT and ESG, and these contradictory research findings largely stem from the fragmentation and limitations of theoretical perspectives. Existing research often takes a single perspective, focusing one-sidedly on a specific mechanism without simultaneously considering both the motivation and capability aspects of how DT affects ESG performance, leading to different explanations for the same phenomenon.

To resolve this theoretical contradiction, this study breaks through two key limitations of existing literature: First, although previous research has confirmed the positive relationship between DT and ESG performance, theoretical mechanism explanations remain scattered: Guo and Pang [10] focus on green innovation and media attention mechanisms; Yang et al. [14] emphasize green innovation, information transparency, and corporate governance; Lu et al. [11] stress internal control and green innovation; Su et al. [12] concentrate on three dimensions of dynamic capabilities; while Li et al. [13] explore risk-taking, organizational conflicts, and managerial overconfidence. While these studies each propose valuable mechanisms, they lack a systematic theoretical framework to organically integrate these dispersed mechanisms. Therefore, this study innovatively constructs a “capability-motivation” analytical framework based on resource dependence theory and agency theory. This framework not only systematically summarizes the scattered mechanisms proposed in existing literature but, more importantly, provides a unified theoretical explanation: from a resource dependence perspective, DT enhances companies’ capabilities to implement ESG by providing technological and resource advantages. From an agency theory perspective, DT fundamentally changes management’s intrinsic motivation to advance sustainable development by reducing information asymmetry and agency costs. Furthermore, although Li et al. [13] preliminarily discovered that the enterprise life cycle moderates the relationship between DT and ESG performance, they failed to deeply analyze the operational mechanisms behind this heterogeneity at the theoretical level. Through the “capability-motivation” framework, this study systematically elaborates how differences in resource endowments, strategic priorities, and internal and external governance pressures exhibited by enterprises at different life cycle stages affect the pathways and intensity of DT’s promotion of ESG performance, thereby filling the theoretical gap in existing research and providing more targeted guidance for enterprises to formulate digital and sustainable development strategies at different development stages.

Given the research gaps mentioned above, this paper systematically examines the influence mechanisms of digital transformation (DT) on corporate ESG performance based on data from Chinese A-share listed companies from 2010–2022, with special attention to heterogeneous characteristics from a life cycle perspective. The empirical test results reveal four core findings: First, DT has a significant promotional effect on corporate ESG performance, a conclusion that remains highly stable after rigorous robustness tests using instrumental variable method, system GMM estimation, and a series of other checks. Second, mechanism analysis reveals that DT influences corporate ESG performance through the dual pathways of resource effect and governance effect. Third, life cycle-based analysis shows that DT’s promotional effect on corporate ESG exhibits obvious stage differences—this positive impact is more significant in mature and declining companies but not significant in growth-stage companies. Finally, in state-owned enterprises, DT’s promotional effect on corporate ESG is stronger.

This research makes theoretical contributions in three main areas based on existing literature. First, this paper innovatively constructs an “capability-motivation” analytical paradigm based on resource dependency theory and agency theory, systematically analyzing the internal logic of how digital transformation promotes enterprise ESG performance. Unlike the fragmented characteristics of previous research in explaining this relationship [10–12,14], this study empirically verifies that DT influences ESG performance through the dual pathways of resource effect (capability dimension) and governance effect (motivation dimension). This dual theoretical framework not only integrates the scattered findings of existing research but also provides a more complete explanatory framework for understanding the complex relationship between DT and ESG performance. Second, based on Adizes’s [16] life cycle theory, this study deepens the theoretical understanding of the moderating effect of the corporate life cycle. Although Li et al. [13] have identified the moderating role of the life cycle on the relationship between DT and ESG, they failed to provide a systematic theoretical explanation. Through the “capability-motivation” analytical paradigm, this study clarifies the systematic changes in resource conditions and intrinsic motivations faced by enterprises at different life cycle stages. This theoretical framework not only supplements the theoretical gap in Li et al.’s [13] research but also deepens our understanding of the relationship between DT and ESG from a life cycle perspective. Finally, by introducing ownership nature as a key contextual variable, this research deepens the understanding of the moderating effect of property rights based on the “capability-motivation” framework. Unlike previous literature that singly explores the impact of ownership on the relationship between DT and ESG, this study analyzes how state-owned enterprise managers’ multiple goal orientations enhance the intrinsic motivation for digital transformation to promote ESG, and how their financing advantages strengthen DT’s impact on ESG. This analytical framework more systematically explains how property rights nature shapes enterprises’ motivation and capability to pursue ESG and thereby improves ESG performance.

## 2. Theoretical analysis and research hypotheses

### 2.1. DT and corporate ESG performance

As global sustainability challenges such as climate change, widening social wealth disparities, and infectious diseases become increasingly severe, businesses are expected to integrate sustainability concepts into their operational decisions, providing solutions to macro-social problems through sustainable practices at the micro level [17]. However, corporate ESG practices face significant externality problems, often leading to insufficient investment at socially optimal levels. Traditional corporate theory advocates that increasing private profit is the only social responsibility of businesses, arguing that imposing other responsibility requirements on businesses is not only impractical but may also erode the effective functioning of the market economy [18]. This narrow view of corporate responsibility is no longer sufficient to meet the expectations of diverse stakeholders in contemporary social environments, but this theoretical tradition continues to influence the decision-making logic of many businesses. From a practical perspective, many businesses face prohibitively high ESG development costs due to resource constraints, insufficient technical capabilities, and information asymmetry with stakeholders. In recent years, the deep integration of digital technology with the real economy has provided new potential pathways for businesses to break through ESG dilemmas [11,15]. Digital technologies embody the digital transformation (DT) of corporate organizational management and production models. Technologies such as big data, cloud computing, blockchain, and artificial intelligence have already played important roles in clean production, recycling, energy conservation, emission reduction, and carbon reduction [19,20]. For example, IBM’s supply chain transparency system built using blockchain technology helps businesses track the environmental impact throughout a product’s entire lifecycle; Alibaba has significantly reduced carbon emissions by optimizing logistics routes through cloud computing and AI technologies.

Specifically, from the perspective of corporate motivation to improve ESG, agency cost theory indicates that agency conflicts arising from the separation of company ownership and control may cause managers to act against shareholders’ optimal interests [21]. In the ESG context, management is often unwilling to make ESG investments that create long-term value but have higher short-term costs due to short-term performance pressures and career concerns [22]. DT significantly reduces agency costs related to ESG practices through multiple channels, thereby promoting improved corporate ESG performance. First, DT strategies drive disruptive changes in management models, reshaping management systems and optimizing organizational structures through digital technology, effectively reducing management costs, eliminating redundant processes, and reducing information transmission barriers [23,24]. In this process, the relationship between shareholders and management undergoes positive changes, making managers more inclined to consider the long-term value creation of ESG strategies, thereby reducing the company’s agency costs. Second, research by He and Sheng [25] found that bank digitalization enables banks to use advanced technology to quickly extract valuable information from massive data, providing comprehensive support for pre-loan screening, loan decision-making, and post-loan management, thus constraining corporate executive behavior and alleviating agency conflicts between managers and shareholders. Third, DT increases the transparency of corporate business processes through the construction of data centers and information systems, making management’s ESG decisions and actions more visible. This transparency reduces opportunities for management’s speculative behavior in environmental responsibility, social impact, and corporate governance. This data-driven monitoring significantly reduces internal monitoring costs and effectively constrains self-interested behavior by management [26]. When management knows their ESG practices are continuously monitored, they are more likely to take actions that align with sustainable development and long-term shareholder interests. Additionally, DT companies typically receive high attention from external stakeholders such as investors, financial analysts, media, and government [27]. This enhanced external supervision forms additional governance mechanisms that further alleviate agency conflicts and mitigate the impact of managerial short-sighted behavior. All these factors are conducive to improving corporate ESG performance. Finally, managerial myopia is an important source of agency costs, with myopic managers focusing excessively on short-term profits and unwilling to bear the initial costs of ESG investments [28]. DT reduces agency costs caused by myopic behavior by enhancing transparency and supervision, forcing managers to focus more on the long-term value of the enterprise rather than short-term performance, thereby creating a more favorable internal environment for ESG practices.

From the perspective of companies’ ability to improve ESG performance, implementing ESG strategies requires adequate resource guarantees, and a company’s ability to obtain resources directly determines its ESG performance level. Resource dependence theory points out that a company’s survival and development essentially depend on its ability to acquire and control key external resources [29]. In this context, DT alleviates corporate financing constraints and optimizes resource acquisition capabilities while significantly promoting ESG performance, forming a synergistic mechanism. Information asymmetry in the market makes it difficult for companies to fully grasp financing information and find effective financing paths, ultimately facing financing constraints. Based on new-generation digital technologies, DT improves corporate information transparency and reduces information asymmetry with financial institutions [30], thereby alleviating financing limitations. DT can quickly collect and organize relevant information from inside and outside the enterprise, transforming unstructured data into structured data, enabling companies to transmit key information such as operating conditions and development potential to financial institutions in a timely and comprehensive manner. This improvement in information transparency directly enhances the company’s ability to implement ESG strategies. Financial institutions can more comprehensively and accurately understand companies undergoing digital transformation, reducing the degree of information asymmetry and improving financing availability. Research by Li et al. [31] shows that digital technology improves the level of corporate credit and financial information disclosure, enabling financial institutions to more quickly identify high-quality companies and enhance their willingness to lend. At the same time, companies can use the information sharing advantages brought by digitalization to obtain financing information more quickly, improve information asymmetry problems, alleviate financing constraints, and provide financial guarantees for ESG investments. In addition, companies that have completed digital transformation often possess stronger data mining and analysis capabilities, which serve as positive signals to investors, indicating that these companies can more precisely identify market opportunities and ESG-related risks, enhancing investor confidence. Research by Wang et al. [32] confirms that the application of digital technology has alleviated information asymmetry and created a more favorable financing environment. As DT becomes a social consensus, highly digitalized companies not only receive policy recognition but also generate higher expectations among investors, becoming investment hotspots, ensuring long-term solvency, and reducing financial distress risks. This financial advantage directly enhances companies’ ability to improve ESG performance.

Based on the above, we propose Hypothesis 1 and Hypothesis 2:

***Hypothesis 1: DT can promote corporate ESG performance***
***Hypothesis 2: DT improves corporate ESG performance by alleviating agency problems and financing constraints***

### 2.2. The moderating effect of corporate life cycle

Corporate life cycle theory indicates that companies in different development stages exhibit systematic differences in strategic priorities, resource allocation, and stakeholder expectations [16,33,34]. This study proposes that the corporate life cycle moderates the promoting effect of DT on ESG performance. Specifically, compared to companies in the growth stage, DT in mature and declining companies has a more significant positive impact on ESG performance, as analyzed below.

From the motivation dimension under agency theory perspective, companies in the growth stage face dual pressures of rapid expansion and resource constraints [35], with shareholders and management having highly aligned interests regarding short-term growth objectives, resulting in relatively smaller agency conflicts. Meanwhile, growth-stage companies typically have more concentrated ownership structures, with management closely supervised by founders or venture capital institutions, and performance evaluation systems primarily built around market share growth and profitability improvement. This agency relationship structure leads management to prioritize allocating limited resources to activities that directly promote short-term performance, viewing DT as a tool to improve operational efficiency rather than to promote corporate ESG performance. In contrast, mature companies typically have more dispersed ownership structures and face more complex agency problems, manifested as intensified information asymmetry between shareholders and management and expanded managerial discretion [36]. In this situation, diversified shareholder groups, especially institutional investors, tend to view ESG performance as an important indicator for evaluating a company’s long-term value creation capability. Therefore, management in mature companies has more motivation to strategically integrate DT with ESG goals, reducing agency costs and enhancing organizational legitimacy by meeting diverse shareholders’ expectations for long-term sustainable development. For companies in the decline stage, shareholders and management face an organizational survival crisis, causing a qualitative change in the characteristics of their agency relationship, with a sharp rise in management tenure risk. At this stage, management urgently needs to rebuild investor confidence and market trust through strategic transformation. ESG becomes a key tool for management to demonstrate strategic renewal capabilities and reshape organizational reputation [37], which helps alleviate investor doubts about management capabilities and reduce agency risk premiums.

Based on resource dependency theory perspective, from the capability dimension, although growing companies typically possess stronger innovative vitality and market sensitivity, they face constraints from scarce resources and unbalanced organizational capability development. Their digital infrastructure is still in the construction process, and limited resources are often concentrated on profit growth objectives [38]. In contrast, mature companies have developed comprehensive organizational structures, standardized processes, and diversified resource reserves [36], demonstrating stronger cross-domain capability integration and strategic execution. Mature companies, by integrating diverse resource advantages, can achieve both optimization of resource allocation and enhancement of resource synergy effects. These enterprises utilize accumulated surplus resources to provide a buffer for ESG investments, while simultaneously reducing unit input costs through economies of scale, forming unique resource competitive advantages. Additionally, the stable external relationship networks already established by mature enterprises offer broader resource acquisition channels for digital transformation and ESG development, promoting cross-organizational knowledge sharing and resource complementarity. Although declining enterprises show an overall downward trend in resources and capabilities [38,39], under market trust and reputation crises, they often use ESG as a strategic tool to salvage corporate image and rebuild stakeholder trust. Consequently, they are willing to prioritize limited resources for digital projects that can rapidly improve ESG performance, hoping to reshape their market image and social value through sustainable development practices [37]. Based on the above analysis, we propose hypothesis 3:

***Hypothesis 3: Compared to growing enterprises, the positive impact of DT on ESG performance is more significant in mature and declining enterprises.***

### 2.3. The moderating effect of ownership nature

The nature of enterprise ownership, as an important institutional factor, influences organizational decision frameworks and resource distribution approaches. This research believes that compared to privately-held entities, the enhancing effect of DT on ESG performance is more significant in state-owned enterprises. This assertion can be analyzed from the dimensions of motivation and capability.

From the motivational perspective, government-controlled and private sector organizations have fundamental differences regarding their ESG strategic approaches. Public sector entities not only carry financial obligations but also shoulder important social responsibilities and policy implementation functions [40]. This special positioning gives them unique advantages and motivation in the field of sustainable development. The multiple goal orientation of state-owned enterprises makes them more willing to incorporate ESG practices into their strategic core and view them as an important source of political legitimacy, which is reflected in their organizational structure, resource allocation, and business decisions [41,42]. When state-owned enterprises implement digital transformation, they tend to proactively integrate ESG factors into their digital strategic framework, viewing digital technology as a powerful tool for achieving sustainable development goals. Managers of state-owned enterprises focus more on broad stakeholder approval rather than purely short-term financial performance. Therefore, their digital investment decisions are more inclined to balance social value and economic value, willing to bear longer investment return cycles, and place greater emphasis on the weight of non-financial performance indicators in evaluation systems [43,44]. In contrast, ESG practices in non-state-owned enterprises are often driven more by market pressure and economic incentives, with their digital strategies focusing more on improving operational efficiency and financial performance. With limited resources, non-state-owned enterprises may tend to selectively implement projects that can bring obvious economic returns.

From the capability perspective, state-owned enterprises provide solid guarantees for the integration of digital transformation and ESG practices through their rich resource base and strong institutional support. State-owned enterprises can obtain sufficient government funding and policy support for digital infrastructure construction, effectively alleviating resource constraints. As implementers of national strategies, these enterprises have stable financing channels. Their close connections with government and financial institutions result in lower financing costs and fewer difficulties [45,46], enabling them to continuously invest in improving ESG performance. Additionally, state-owned enterprises occupy advantageous positions in acquiring key resources [47,48]. They often have priority access to cutting-edge digital technologies and high-quality professional talent, which not only enhances the effectiveness of digital transformation but also ensures the long-term driving force for sustainable enterprise development. In contrast, non-state-owned enterprises often face more limitations and challenges in these aspects.

Based on the above analysis, we propose hypothesis 4:

***Hypothesis 4: Compared to private enterprises, the positive impact of DT on ESG performance is more significant in state-owned enterprises.***

## 3. Research design

### 3.1. Sample selection and data sources

This study selects Chinese A-share listed companies from 2010 to 2022 as research subjects, based on China’s unique position and representativeness in the global digital economy development. As the world’s second-largest economy, Chinese enterprises demonstrate diverse practical pathways and rich evolutionary models in their digital transformation process [49], providing an ideal sample for studying the relationship between digitalization and ESG performance. China has achieved significant accomplishments in digital technology applications, with multiple indicators such as mobile payment penetration rate, e-commerce scale, and digital infrastructure construction ranking among the world’s top, which gives Chinese enterprises’ DT distinctive characteristics and high representativeness. Simultaneously, the Chinese government has constructed a policy framework for the coordinated advancement of digitalization and sustainable development through the implementation of national strategies such as “Internet+”, “Digital China”, and “Carbon Peak and Carbon Neutrality”, creating a unique environment for the deep integration of DT and ESG practices. Furthermore, as the largest developing country, China’s DT experience has important reference value for other emerging economies, and the research results help reveal the universal patterns and special mechanisms of digitalization in promoting sustainable development.

To ensure the reliability and validity of empirical research results, this study conducted systematic screening and processing of the initial sample: First, we eliminated financial industry company samples because financial enterprises have special financial structures and regulatory environments that might affect the universality of results [50,51]. Second, to ensure data continuity and completeness, we excluded companies that were suspended from listing or delisted during the sample period, eliminating data bias potentially introduced by abnormal business operations. Furthermore, we removed samples of ST, *ST, and PT type companies, as these companies typically face financial distress or significant operational abnormalities that could unnecessarily interfere with research results [52]. Finally, we eliminated samples with missing indicators, ensuring the completeness of analysis variables and the accuracy of research results. After the above rigorous screening, this study ultimately obtained balanced panel data with 22,988 company-year observations. The enterprise digitalization indicators in the study were mainly constructed through textual analysis of listed companies’ annual reports, corporate financial data was sourced from the CSMAR database, and ESG performance data came from the Wind Financial database.

### 3.2. Variable definitions

#### 3.2.1. Explained variable: Enterprise Environmental, Social and Governance Performance (ESG).

When conducting ESG-related research, clearly defining core concepts is a key step in ensuring research accuracy. ESG disclosure and ESG performance are two interrelated but distinctly different concepts. ESG disclosure focuses on the transparency and quality of ESG-related information published by enterprises [53], while ESG performance directly reflects the actual actions and effectiveness of enterprises in environmental, social, and governance aspects [54]. This conceptual distinction is particularly important for this study because we need to conduct analysis based on accurate ESG performance assessments, rather than solely relying on the level of information disclosure by enterprises.

Based on this conceptual framework, this study needs to select appropriate indicators to accurately measure the ESG performance of Chinese enterprises. With sustainable investment principles gaining traction globally, numerous evaluation frameworks have emerged internationally, which differ in assessment criteria, reference indicators, and coverage. After comparative analysis, this paper selects the Huazheng ESG rating indicator to measure corporate ESG practices. This is because compared to other rating systems, Huazheng ESG ratings have significant advantages in terms of coverage and update frequency for Chinese enterprises. Huazheng ESG ratings reference international mainstream methods and practical experiences, while combining Chinese national conditions and capital market characteristics, providing the market with rating results in three dimensions: environmental, social, and corporate governance for China’s A-share listed companies. Currently, Huazheng ESG rating data has been widely recognized and applied in business and academic circles [11,55].

Huazheng ESG ratings are divided into nine grades. This paper assigns values from 1 to 9 to corporate ESG ratings from low to high, with larger values representing higher levels of ESG performance. In the main regression analysis, we use the average of the enterprise’s quarterly ESG rating indicators as the measurement standard for corporate ESG performance to comprehensively reflect the overall performance of enterprises during the study period. In robustness tests, we adopt the median of ESG quarterly ratings to measure corporate ESG performance, reducing the impact of potential abnormal fluctuations between quarters, thereby ensuring the robustness of research conclusions. This measurement method enables us to more accurately capture the actual ESG performance of enterprises, rather than merely focusing on their disclosure behavior, thus providing a reliable data foundation for subsequent research.

#### 3.2.2. Explanatory variable: Enterprise Digital Transformation (DT).

Text analysis methods are now widely applied in variable measurement [56]. Drawing on previous research, we also use text analysis to measure DT. In the selection of characteristic words, we referenced the research of Wu et al. [57], dividing DT into two levels: “underlying technology application” and “technology practice application.” Underlying technology application includes artificial intelligence, blockchain, cloud computing, big data, and other technologies, which constitute the core technical architecture of DT, mainly focusing on the digital conversion and upgrade of internal production operations and management models of enterprises. Technology practice application focuses on the innovative integration of digital technology with complex business scenarios, gradually expanding from back-end technological empowerment to front-end market application scenarios, reflecting the deep development path of DT from technological innovation to business transformation. In practical operation, we collected annual reports of A-share listed companies from Shanghai and Shenzhen stock exchanges through Python web crawlers, extracted text content using the Java PDFbox library, and adopted the DT keywords used in Wu et al.‘s [57] research to form a structured feature word map. During the word frequency statistical analysis process, we eliminated expressions containing negation words such as “no,” “none,” “not,” etc., as well as DT vocabulary not related to the company itself, ensuring the accuracy and validity of the data. Since word frequency data has typical “right-skewed” characteristics, we applied logarithmic processing to the finally collected word frequencies, constructing a more reasonable comprehensive DT indicator.

#### 3.2.3. Agency costs and financing constraints.

This paper selects two types of agency costs as mediating variables for corporate internal governance. Referencing the research of Ang et al. [58], the first type of agency cost (Agent1) is measured using the ratio of administrative expenses to operating revenue, which reflects potential excessive consumption and inefficient expenditure behaviors by management during company operations. Based on the research of An et al. [59], we use the ratio of other net receivables to operating revenue to calculate the second type of agency cost (Agent2), which mainly measures the degree of resource occupation by major shareholders and reflects the agency conflicts between major and minor shareholders. Additionally, this study refers to the method of Wu and Huang [60], using the FC index as a proxy variable for corporate financing capability. This index reflects the difficulty level of obtaining external funds by comprehensively considering factors such as enterprise size, age, dividend payments, and cash holdings.

#### 3.2.4. Enterprise life cycle.

This study adopts the cash flow combination classification method proposed by Dickinson [34], combined with the five-stage enterprise life cycle theory (introduction period, growth period, maturity period, elimination period, and decline period) constructed by Gort and Klepper [61], to scientifically classify the life cycle stages of Chinese listed companies. The conceptualization and measurement method selection of the enterprise life cycle has an important impact on research results. The cash flow combination method is based on the inherent logic of corporate financial activities, precisely identifying the development stage of an enterprise by analyzing the positive and negative combination patterns of the net cash flows from three major categories: operating activities, investing activities, and financing activities. Compared to traditional classification methods that rely on single indicators (such as enterprise size, age, or growth rate), the cash flow combination method can more comprehensively capture the dynamic process of enterprise resource acquisition and allocation, effectively avoiding classification biases that might result from a single dimension. Considering research needs and sample characteristics, this study appropriately simplified the five-stage model, merging the introduction period and growth period into a growth period, and combining the elimination period and decline period into a decline period.

In the selection of enterprise life cycle measurement methods, academia has multiple effective approaches. The “Comprehensive Score Discrimination Method” applied by Iftikhar et al. [62] provides a classification perspective based on relative positions within an industry. This method ranks enterprises by score and categorizes them as growth-type, mature-type, and declining-type. This method has certain advantages in capturing the relative development status of enterprises within an industry. The cash flow combination method adopted in this study is based on the identification of an enterprise’s own financial activity patterns, directly reflecting the strategic choices of enterprises in resource acquisition, investment expansion, and financing strategies through cash flow signals, which has a high theoretical fit with the issues this research focuses on. Both methods have value in different research contexts. Based on the research questions, this study selected the cash flow combination method as the measurement tool for enterprise life cycles. Table 1 presents in detail the cash flow characteristic combinations of each stage of the enterprise life cycle and their theoretical explanations.

**Table 1:** Enterprise life cycle classification.

Cash Flow	Introduction Period	Growth Period	Maturity Period	Elimination Period	Elimination Period	Elimination Period	Decline Period	Decline Period
Operating Cash Flow	–	+	+	–	+	+	–	–
Investment Cash Flow	–	–	–	–	+	+	+	+
Financing Cash Flow	+	+	–	–	+	–	+	–

#### 3.2.5. Control variables.

This paper builds upon prior literature [19,63,64] and incorporates the following control variables across three categories: enterprise characteristics, financial status, and corporate governance status. Enterprise size (Size) is calculated as the logarithm of the company’s total assets at year-end. Debt-to-asset ratio (Lev) represents the proportion of the firm’s total liabilities relative to its total assets at year-end. Return on assets (ROA) is calculated by dividing net profit by total assets at year-end. Cash flow ratio (Cashflow) measures the relationship between net cash flow from operations and total assets. Institutional investor shareholding ratio (INST) indicates the percentage of shares owned by institutional investors out of total outstanding shares. Shareholding ratio of the largest shareholder (Top1) represents the percentage of total share capital held by the primary shareholder. Board independence (Indep) is calculated as the percentage of independent members on the board of directors. Equity balance degree (Balance) is determined by comparing the combined shareholding percentages of the second through fifth largest shareholders to that of the primary shareholder. Enterprise internationalization degree (FSTS) is evaluated using the ratio of overseas revenue to operating revenue. Enterprise R&D intensity (RD) is assessed by examining R&D expenditure as a percentage of operating revenue. Table 2 provides comprehensive definitions of all variables utilized in this study.

**Table 2:** Variable definitions.

Name	Symbol	Definition
Corporate ESG	ESG	ESG rating on a scale of 1–9, with higher scores indicating better corporate ESG performance.
Enterprise digitalization level	DT	Measured using text analysis methods through Python web crawlers to extract digitalization-related keywords from annual reports. The frequency of these keywords is normalized by the total length of MD&A segments to construct a comprehensive digitalization metric.
Agency Cost (Type 1)	Agent1	Ratio of administrative expenses to operating revenue, reflecting potential excessive consumption and inefficient expenditure by management.
Agency Cost (Type 2)	Agent2	Ratio of other net receivables to operating revenue, measuring resource occupation by major shareholders and reflecting agency conflicts between major and minor shareholders.
Financing Constraints	FC	A comprehensive index reflecting the difficulty level of obtaining external funds, considering factors such as enterprise size, age, dividend payments, and cash holdings.
Firm size	Size	Natural logarithm of total assets at the end of the year
Asset liability ratio	Lev	Ratio of total liabilities to total assets.
Firm profitability	ROA	Ratio of net income to total assets, measuring firm profitability.
Independent boards	Indep	Proportion of independent directors to the total number of board members.
Cash flow ratio	Cashflow	Ratio of net cash flows from operating activities to total assets at the end of the year.
Institutional investor	INST	Percentage of shares held by institutional investors relative to total outstanding shares.
Equity balance	Balance	Ratio of the combined shareholding of the second to fifth largest shareholders to the shareholding of the largest shareholder.
The largest shareholder	Top1	Percentage of shares held by the largest shareholder relative to total share capital.
R&D Intensity	RD	Ratio of R&D expenditure to operating revenue
Internationalization Degree	FSTS	Ratio of overseas revenue to total operating revenue

### 3.3. Econometric models

#### 3.3.1. Baseline regression model.

This study constructs the following baseline regression model to test the impact of DT on enterprise ESG performance.


$$ESG_{i,t}=\alpha_{0}+\alpha_{1}DT_{i,t}+\sum \psi_{n}Controls+\lambda_{i}+\eta_{t}+\varepsilon_{i,t}$$ (1)

In this equation, subscript i represents individual enterprises while t represents the year. The explained variable ESG represents the environmental, social, and governance performance of enterprises. The explanatory variable DT represents the level of digital transformation. Controls represents control variables. λ_i_ and η_t_ represent enterprise fixed effects and year fixed effects respectively. ε_it_ represents the error term. If DT significantly improves corporate ESG, then α_1_ should be significantly positive.

#### 3.3.2. Mechanism testing model.

To deeply explore the mechanism between DT and ESG performance, this study constructs a mediation effect model based on the baseline regression model for testing. The mediation effect test follows these steps. First, after confirming that the regression coefficient α1 of DT on enterprise ESG performance is significant, establish a regression equation (2) of DT on the mediating variable. Second, construct a comprehensive regression equation (3) that includes both DT and the mediating variable on enterprise ESG performance. By evaluating the significance and magnitude of parameters γ_1_, β_1_, and β_2_, we can determine the mediating role played by the mediating variable in the relationship between DT and ESG performance.


$$M_{i,t}=\alpha_{0}+\gamma_{1}DT_{i,t}+\sum \psi_{n}Controls+\lambda_{i}+\eta_{t}+\varepsilon_{i,t}$$ (2)

$$ESG_{i,t}=\beta_{0}+\beta_{1}DT_{i,t}+\beta_{2}M_{i,t}+\sum \psi_{n}Controls+\lambda_{i}+\eta_{t}+\varepsilon_{i,t}$$ (3)

## 4. Results

### 4.1. Descriptive statistics

This study utilizes data from Chinese A-share listed companies from 2010 to 2022, covering a total of 22,988 observations. Descriptive statistics show that the ESG performance of sample companies is measured in two ways: the quarterly mean ESG rating of 4.1680 (standard deviation 0.8200) and the quarterly median ESG rating of 4.1683 (standard deviation 0.8544) (Table 3). These two indicators are close in value and both at a medium level, indicating that companies’ ESG ratings remain relatively stable across quarters. The mean DT indicator is 1.4228 (standard deviation 1.3604), reflecting significant differences in the digitalization process among companies. Regarding mediating variables, the mean of financing constraints (FC) is 0.4628 (standard deviation 0.2824), indicating that the sample companies generally face medium-level financing constraints, but with considerable variations among companies. The mean of the first type of agency cost (Agent1) is 0.0851 (standard deviation 0.0647), indicating that management expenses account for an average of 8.51% of operating revenue; the mean of the second type of agency cost (Agent2) is 0.0319 (standard deviation 0.0685), reflecting that the net amount of other receivables accounts for an average of 3.19% of operating revenue. The standard deviations of these two types of agency costs are relatively high, indicating significant differences in internal governance efficiency among different companies. Regarding control variables, the sample companies have a total asset size (logarithmic value) of 22.3074, an average asset-liability ratio of 42.84%, and average return on assets and operating cash flow ratios of 4.06% and 4.82%, respectively. These indicators collectively suggest that the sample companies are in good overall financial condition. In terms of corporate governance characteristics, the average proportion of independent directors on the board reaches 37.63%, institutional investor shareholding is 44.70%, the average shareholding ratio of the largest shareholder is 34.18%, and the equity balance degree is 0.7306, reflecting a generally concentrated ownership structure among sample companies. Additionally, companies have an average internationalization degree of 13.57% and R&D intensity of 4.52%. The statistical characteristics of these control variables are generally consistent with existing literature [11,52].

**Table 3:** Descriptive statistics.

Variable	Obs	Mean	Std. dev.	Min	Max
ESG_mean	22,988	4.1680	0.8200	1.5000	6.0000
ESG_med	22,988	4.1683	0.8544	1.5000	6.0000
DT	22,988	1.4228	1.3604	0.0000	5.2204
FC	22,988	0.4628	0.2824	0.0042	0.9636
Agent1	22,988	0.0851	0.0647	0.0070	0.7247
Agent2	22,988	0.0319	0.0685	0.0001	1.0431
Size	22,988	22.3074	1.2852	19.9588	26.1903
Lev	22,988	0.4284	0.1953	0.0495	0.8689
ROA	22,988	0.0406	0.0631	−0.2307	0.2278
Cashflow	22,988	0.0482	0.0647	−0.1445	0.2411
Indep	22,988	0.3763	0.0536	0.3333	0.5714
INST	22,988	0.4470	0.2522	0.0028	0.9291
Top1	22,988	0.3418	0.1454	0.0843	0.7313
Balance	22,988	0.7306	0.5943	0.0344	2.8682
FSTS	22,988	0.1357	0.2144	0.0000	0.8999
RD	22,988	0.0452	0.0477	0.0002	0.2978

### 4.2. Baseline regression results

Hypothesis 1 proposes that DT enhances corporate ESG performance. According to the regression results in Table 4, the relationship between DT and corporate ESG performance demonstrates a robust positive correlation across all three models. In the first column’s baseline model, the DT coefficient is 0.0392, significant at the 1% level, indicating that DT has a positive promoting effect on ESG performance. In the second column, after introducing financial-related variables, the DT coefficient decreases to 0.0228 but remains highly significant, suggesting that the positive effect of DT on ESG remains robust after controlling for financial factors. In the third column, after further incorporating governance-related variables, the DT coefficient slightly increases to 0.0243 and passes the significance test, further confirming a robust positive relationship between DT and ESG performance. The three models consistently indicate that the higher a company’s DT level, the better its ESG performance, with this association remaining valid after adding different types of control variables. From an economic significance perspective, taking the third column model as an example, when DT increases by one standard deviation (1.3604), a company’s ESG performance (ESG_mean) is expected to increase by 0.0403 standard deviations. Among the control variables, company size, return on assets, proportion of independent directors, shareholding ratio of the largest shareholder, and R&D intensity all show significant positive correlations with ESG performance, while asset-liability ratio and operating cash flow ratio show significant negative correlations. The adjusted R-squared for each model ranges between 0.476–0.497, indicating that the models have good explanatory power.

**Table 4:** Benchmark regression results.

Variables	(1)	(2)	(3)
	ESG_mean	ESG_mean	ESG_mean
DT	0.0392***	0.0228***	0.0243***
	(0.008)	(0.008)	(0.00810)
Size		0.2240***	0.229***
		(0.018)	(0.0188)
Lev		−0.7626***	−0.751***
		(0.067)	(0.0673)
ROA		0.6651***	0.644***
		(0.131)	(0.131)
Cashflow		−0.3600***	−0.353***
		(0.097)	(0.0966)
Indep			1.025***
			(0.172)
INST			−0.107
			(0.0681)
Top1			0.483***
			(0.144)
Balance			0.0410
			(0.0277)
FSTS		−0.0219	−0.0187
		(0.062)	(0.0622)
RD		1.0930***	1.099***
		(0.294)	(0.293)
Constant	4.1123***	−0.5913	−1.242***
	(0.012)	(0.401)	(0.414)
Observations	22,988	22,988	22,988
Adjusted R-squared	0.476	0.495	0.497
Firm FE	YES	YES	YES
Year FE	YES	YES	YES

### 4.3. Instrumental variable method

Considering the potential endogeneity concerns between DT and ESG performance, including possible reverse causality (firms with strong ESG ratings may prioritize DT investments) and omitted variable bias (factors such as management competence, corporate culture, etc., which could simultaneously influence both digital transformation initiatives and ESG outcomes), this study employs the industry-year level DT average as an instrumental variable and utilizes two-stage least squares (2SLS) methodology to establish more reliable causal inferences.

The instrumental variable’s validity has undergone comprehensive examination. The Kleibergen-Paap rk LM statistic value of 220.641 (p = 0.000) rejects the under-identification null hypothesis. Additionally, the Kleibergen-Paap rk Wald F statistic value of 399.007 substantially exceeds Staiger and Stock’s [65] proposed critical threshold of 10, rejecting the weak instrument hypothesis. These assessments confirm our chosen instrumental variable satisfies both relevance and exogeneity requirements.

The results in column 2 of Table 5 indicate that DT, after being treated with instrumental variables, has a significant positive impact on ESG performance, with a coefficient of 0.0754 (p < 0.1). Through the instrumental variable method, the research further confirms a causal relationship between DT and ESG performance, namely, DT indeed drives the improvement of corporate ESG performance.

**Table 5:** Instrumental variable and system GMM test results.

Variables	(1)	(2)	(3)
	DT	ESG_mean	ESG_mean
L.ESG_mean			0.8653***
			(0.027)
DT		0.0754*	0.0393**
		(0.040)	(0.016)
Size	0.2258***	0.2154***	0.0244*
	(0.022)	(0.021)	(0.014)
Lev	−0.1513*	−0.7462***	−0.3655***
	(0.085)	(0.068)	(0.091)
ROA	−0.0621	0.6523***	0.1084
	(0.140)	(0.131)	(0.169)
Cashflow	−0.0572	−0.3515***	−0.0927
	(0.103)	(0.096)	(0.158)
Indep	−0.2074	1.0359***	0.3540***
	(0.177)	(0.173)	(0.124)
INST	−0.1408	−0.1007	0.0967
	(0.090)	(0.068)	(0.071)
Top1	−0.3303*	0.5050***	−0.1391
	(0.190)	(0.145)	(0.353)
Balance	0.0079	0.0412	0.0150
	(0.036)	(0.028)	(0.102)
FSTS	−0.1872**	−0.0062	−0.0199
	(0.085)	(0.063)	(0.215)
RD	0.9188**	1.0250***	−2.8309**
	(0.415)	(0.292)	(1.099)
IV	0.5869***		
	(0.029)		
Constant			−0.0560
			(0.411)
Observations	22,988	22,988	16,434
Kleibergen-Paap rk LM	220.641		AR(1)=0.000; AR(2)=0.974
Kleibergen-Paap rk Wald F	399.007		Hansen = 0.140

### 4.4. Endogeneity analysis based on system GMM

To further address potential endogeneity issues and test the robustness of results, we follow Bagh et al. [66] by employing the system GMM method for estimation. System GMM effectively handles potential endogeneity problems and dynamic bias by constructing dynamic panel models and utilizing internal instrumental variables. As shown in column 3 of Table 5, the system GMM estimation results still support that DT has a significant positive impact on ESG performance (coefficient of 0.0393, p < 0.05). Notably, the coefficient of the lagged term of ESG_mean reaches 0.8653 (p < 0.001), indicating that corporate ESG performance has significant persistence characteristics, which further confirms the necessity of adopting dynamic panel models. Model diagnostics indicate that the Arellano-Bond test results meet expectations (AR(1) is significant while AR(2) is not significant), and the Hansen test (p = 0.140) supports the overall validity of instrumental variables, ensuring the reliability of estimation results. Overall, the analysis results of the system GMM are consistent with the 2SLS estimates, further supporting the robustness of the conclusion that DT promotes ESG performance.

### 4.5. Robustness tests

#### 4.5.1. Alternative ESG measurement method.

To address potential skewed distribution characteristics or extreme value influences in ESG scores, this study adopts the ESG median indicator (ESG_med) instead of the mean indicator in column 1 of Table 6, aiming to reduce the potential interference of outliers on estimation results. The analysis results show that the DT variable coefficient is 0.0289, remaining highly significant at the 1% level, which thoroughly validates the robustness of the main research findings to changes in ESG measurement methods. As a disturbance-resistant measure of central tendency, the median can more effectively address data skewness problems compared to the mean. The estimation results indicate that the core conclusion of this study does not depend on the choice of ESG measurement methods, and the conclusion that DT promotes ESG performance is robust.

**Table 6:** Robustness tests.

Variables	(1)	(2)	(3)	(4)	(5)	(6)
	ESG_med	ESG_mean	ESG_mean	ESG_mean	ESG_mean	F.ESG_mean
DT	0.0289***	0.0265***	0.0266***	0.0240***		0.0219**
	(0.009)	(0.009)	(0.009)	(0.008)		(0.010)
Size	0.2343***	0.2320***	0.2450***	0.2288***	0.2315***	0.1919***
	(0.020)	(0.019)	(0.021)	(0.018)	(0.024)	(0.024)
Lev	−0.7324***	−0.7831***	−0.7811***	−0.7747***	−0.6422***	−0.6708***
	(0.071)	(0.071)	(0.076)	(0.067)	(0.083)	(0.084)
ROA	0.6703***	0.6051***	0.5480***	0.6989***	0.5883***	1.7936***
	(0.139)	(0.135)	(0.140)	(0.130)	(0.162)	(0.167)
Cashflow	−0.4111***	−0.3411***	−0.2769***	−0.3346***	−0.3424***	−0.0854
	(0.103)	(0.100)	(0.104)	(0.098)	(0.122)	(0.115)
Indep	1.0410***	1.1585***	1.0109***	0.9814***	1.1258***	0.8989***
	(0.178)	(0.180)	(0.197)	(0.169)	(0.193)	(0.189)
INST	−0.1048	−0.1069	−0.1643**	−0.1246*	−0.0654	0.0091
	(0.071)	(0.072)	(0.075)	(0.067)	(0.081)	(0.081)
Top1	0.4280***	0.4825***	0.5457***	0.5619***	0.3052*	−0.0845
	(0.151)	(0.148)	(0.154)	(0.146)	(0.174)	(0.171)
Balance	0.0424	0.0387	0.0419	0.0544**	0.0310	−0.0373
	(0.029)	(0.028)	(0.030)	(0.028)	(0.034)	(0.033)
FSTS	−0.0142	−0.0060	−0.0221	−0.0401	0.0008	0.0566
	(0.066)	(0.064)	(0.070)	(0.061)	(0.073)	(0.075)
RD	0.9040***	1.0173***	1.1475***	1.0849***	1.3058***	0.3632
	(0.312)	(0.301)	(0.347)	(0.291)	(0.374)	(0.376)
L.DT					0.0221**	
					(0.010)	
Constant	−1.3537***	−1.3396***	−1.5830***	−1.2400***	−1.3420**	−0.2247
	(0.437)	(0.425)	(0.456)	(0.399)	(0.528)	(0.526)
Observations	22,988	21,338	18,624	22,988	16,434	16,434
Adjusted R-squared	0.437	0.489	0.495	0.506	0.507	0.513
Firm FE	YES	YES	YES	Firm FE	YES	YES
Year FE	YES	YES	YES	Year FE	YES	YES

#### 4.5.2. Removing samples from the 2015 stock market crash and direct-administered municipalities.

To exclude potential influences of special event shocks and regional heterogeneity factors, we implemented sample adjustment robustness tests. First, we eliminated samples from the 2015 stock market crash period. The results in column 2 of Table 6 show that even after excluding data from this special market environment, the DT coefficient still maintains at 0.0265 and remains highly significant (p < 0.01). Second, based on the research by Chen et al. [67], we excluded samples from direct-administered municipalities including Beijing, Shanghai, Tianjin, and Chongqing, considering that these regions have unique policy environments, stronger regulatory intensity, and leading economic development levels. The results in column 3 of Table 6 indicate that after excluding these samples, the DT coefficient remains stable at 0.0266 with unchanged statistical significance. These two robustness test results confirm the reliability and universality of our research findings, showing that the research conclusions are not affected by special market environments or regional characteristics.

#### 4.5.3. Adding province and year interactive fixed effects.

To more rigorously control for potentially unobserved time-varying factors at the provincial level (such as trajectories of local policy evolution, regional economic development differences, implementation intensity of ESG initiatives in different regions, etc.), we introduced more stringent province-year interaction fixed effects in column 4 of Table 6. This method can effectively absorb common shocks that different provinces may face in different years. The estimation results show that under this more stringent model specification, the DT coefficient remains at 0.0240 and stays significant at the 1% level. The results indicate that even after controlling for more complex fixed effects structures, the positive relationship between DT and ESG performance remains robust, suggesting that this relationship is unlikely to be driven by omitted variables at the provincial level that change over time.

#### 4.5.4. Tests with altered time windows.

To deeply explore the dynamic evolutionary relationship and potential causal direction between DT and ESG performance, we implemented time window variation tests. Column 5 of Table 6 employs a one-period lagged DT indicator (L.DT), with a coefficient of 0.0221 that is significant at the 5% level. This result not only effectively mitigates potential reverse causality issues but also reveals that DT has a persistent time-lag effect on ESG performance, rather than being merely a short-term phenomenon. Additionally, column 6 of Table 6 examines DT’s impact on future ESG performance (F.ESG_mean), with results showing a coefficient of 0.0219 that is significant at the 5% level, strongly corroborating DT’s predictive capability for future corporate ESG performance. This finding further strengthens the evidence chain for a temporal causal relationship between the variables, indicating that DT indeed drives subsequent improvement in corporate ESG performance rather than representing a simple correlation. Collectively, the results of the time window variation tests demonstrate significant cross-period robustness in our research findings.

### 4.6. Mediating effect analysis

Columns one and two of Table 7 reveal the mechanism through which DT promotes ESG performance by mitigating financing constraints. The first column demonstrates that DT significantly reduces enterprise financing constraints (FC), with a coefficient of −0.0046 that is significant at the 1% level, showing that digital transformation effectively lowers funding barriers encountered by companies. The results in the second column verify that financing limitations negatively affect ESG performance, with a coefficient of −0.1240 that is significant at the 5% level, while DT maintains a direct positive influence on ESG metrics with a coefficient of 0.0237 that is significant at the 1% level. These findings indicate that DT not only directly fosters ESG performance but also indirectly strengthens corporate sustainability through the critical mediating factor of reduced financing constraints, creating favorable conditions for organizations to allocate additional resources toward sustainable development initiatives.

**Table 7:** Mediation effect test.

Variables	(1)	(2)	(3)	(4)	(5)	(6)
	FC	ESG_mean	Agent1	ESG_mean	Agent2	ESG_mean
DT	−0.0046***	0.0237***	−0.0012**	0.0228***	−0.0020***	0.0229***
	(0.001)	(0.008)	(0.001)	(0.008)	(0.001)	(0.008)
FC		−0.1240**				
		(0.055)				
Agent1				−1.2677***		
				(0.181)		
Agent2						−0.6915***
						(0.115)
Size	−0.1501***	0.2104***	−0.0144***	0.2108***	0.0013	0.2299***
	(0.004)	(0.021)	(0.002)	(0.019)	(0.002)	(0.019)
Lev	−0.5185***	−0.8154***	−0.0205***	−0.7771***	0.0174**	−0.7391***
	(0.013)	(0.072)	(0.005)	(0.067)	(0.007)	(0.067)
ROA	0.4962***	0.7059***	−0.1748***	0.4228***	−0.1114***	0.5674***
	(0.023)	(0.134)	(0.011)	(0.134)	(0.015)	(0.130)
Cashflow	−0.1454***	−0.3712***	−0.0081	−0.3634***	−0.0148	−0.3634***
	(0.016)	(0.097)	(0.007)	(0.096)	(0.010)	(0.097)
Indep	0.0023	1.0255***	0.0140	1.0430***	0.0098	1.0320***
	(0.025)	(0.172)	(0.010)	(0.172)	(0.014)	(0.172)
INST	−0.1177***	−0.1218*	0.0070	−0.0984	−0.0011	−0.1080
	(0.011)	(0.068)	(0.005)	(0.067)	(0.006)	(0.068)
Top1	0.2611***	0.5152***	−0.0042	0.4776***	−0.0386**	0.4562***
	(0.025)	(0.145)	(0.010)	(0.144)	(0.015)	(0.144)
Balance	0.0354***	0.0454	0.0011	0.0424	−0.0012	0.0402
	(0.005)	(0.028)	(0.002)	(0.028)	(0.003)	(0.028)
FSTS	0.0126	−0.0171	−0.0073	−0.0280	−0.0039	−0.0214
	(0.011)	(0.062)	(0.006)	(0.062)	(0.005)	(0.062)
RD	−0.1070**	1.0856***	0.5308***	1.7718***	0.1379***	1.1942***
	(0.050)	(0.293)	(0.042)	(0.309)	(0.035)	(0.292)
Constant	3.9669***	−0.7503	0.3931***	−0.7437*	0.0076	−1.2368***
	(0.083)	(0.472)	(0.033)	(0.416)	(0.038)	(0.414)
Observations	22,988	22,988	22,988	22,988	22,988	22,988
Adjusted R-squared	0.898	0.497	0.752	0.499	0.465	0.499
Firm FE	YES	YES	YES	YES	YES	YES
Year FE	YES	YES	YES	YES	YES	YES

Columns three and four of Table 7 demonstrate the mechanism through which DT enhances ESG performance by reducing agency costs between shareholders and management. The third column shows that DT significantly reduces Type I agency costs (Agent1), with a coefficient of −0.0012 that is significant at the 5% level. The fourth column further reveals that Type I agency costs have a significant negative impact on ESG performance, with a coefficient of −1.2677 that is significant at the 1% level, while DT maintains a significant positive impact on ESG (coefficient of 0.0228, p < 0.01), indicating that DT indirectly improves enterprise ESG performance by alleviating traditional shareholder-management agency problems, motivating management to focus more on long-term sustainable development rather than short-term performance.

Columns five and six of Table 7 explain the mechanism through which DT enhances ESG performance by improving agency conflicts between majority and minority shareholders. The fifth column indicates that DT significantly reduces Type II agency costs (Agent2), with a coefficient of −0.002 that is significant at the 1% level, reflecting DT’s positive role in alleviating conflicts of interest between controlling shareholders and minority shareholders, possibly through enhancing transparency and corporate governance mechanisms, which suppress tunneling behaviors and related party transactions by major shareholders. The results in the sixth column show that Type II agency costs also have a significant negative impact on ESG performance, with a coefficient of −0.6915 that is significant at the 1% level, while DT’s direct positive impact on ESG remains robust (coefficient of 0.0229, p < 0.01), further confirming the mediating pathway through which DT indirectly promotes ESG performance improvement by addressing equity agency problems.

Following the approach to mediating effects in existing literature [68,69], in order to accurately assess the statistical significance of mediating effects and obtain precise effect size estimates, we employed the bootstrap method for further testing based on traditional regression analysis. Compared to the traditional Sobel test, the bootstrap method does not rely on the assumption of normal distribution of mediating effects and can provide more accurate estimates of mediating effects and their confidence intervals by constructing empirical distributions through repeated sampling. The specific results of the bootstrap analysis are as follows:

Table 8 shows the bootstrap analysis results of DT affecting ESG performance through financing constraints. The analysis indicates that the indirect effect of DT on ESG performance through alleviating financing constraints is 0.0006, significant at the 5% level (p < 0.05), with a 95% confidence interval of [0.0000443, 0.0010838], which does not contain zero, confirming the mediating role of financing constraints in the relationship between DT and ESG performance. The direct effect of DT on ESG performance is 0.0237 (p < 0.01), and the total effect is 0.0243 (p < 0.01). The mediating effect of financing constraints accounts for approximately 2.47% of the total effect, indicating that financing constraints are a significant pathway through which DT influences ESG performance.

**Table 8:** Bootstrap analysis of financing constraints.

Effect Type	Coefficients	Std. Error	P-Value	95% Confidence Interval
Indirect Effect	0.0006	0.0003	0.033	[0.0000443, 0.0010838]
Direct Effect	0.0237	0.0064	0.000	[0.0111237, 0.0362936]
Total Effect	0.0243	0.0064	0.000	[0.0116736, 0.0368717]

Table 9 presents the bootstrap analysis results of DT affecting ESG performance through Type I agency costs (agency problems between shareholders and management). The results show that the indirect effect of DT on ESG performance through reducing Type I agency costs is 0.0015, significant at the 1% level (p < 0.01), with a 95% confidence interval of [0.0003731, 0.0026], which does not contain zero, confirming that Type I agency costs play an important mediating role in the relationship between DT and ESG performance. The direct effect of DT on ESG performance is 0.0228 (p < 0.01), and the total effect is 0.0243 (p < 0.01). The mediating effect of Type I agency costs accounts for approximately 6.17% of the total effect, which is higher than the proportion of the mediating effect of financing constraints, indicating that DT’s improvement of agency problems between shareholders and management represents a more significant indirect pathway.

**Table 9:** Bootstrap analysis of Type 1 agency costs.

Effect Type	Coefficient	Std. Error	P-Value	95% Confidence Interval
Indirect Effect	0.0015	0.0006	0.009	[0.0003731, 0.0026]
Direct Effect	0.0228	0.0064	0.000	[0.0102393, 0.0353329]
Total Effect	0.0243	0.0064	0.000	[0.0116736, 0.0368717]

Table 10 presents the bootstrap analysis results of DT affecting ESG performance through Type II agency costs (agency problems between majority and minority shareholders). The results indicate that the indirect effect of DT on ESG performance through reducing Type II agency costs is 0.0014, significant at the 1% level (p < 0.01), with a 95% confidence interval of [0.000406, 0.0023027], which does not contain zero, confirming the mediating role of Type II agency costs in the relationship between DT and ESG performance. The direct effect of DT on ESG performance is 0.0229 (p < 0.01), and the total effect is 0.0243 (p < 0.01). The mediating effect of Type II agency costs accounts for approximately 5.76% of the total effect, comparable to the proportion of the mediating effect of Type I agency costs, indicating that the improvement of agency problems between majority and minority shareholders is also an important indirect pathway through which DT influences ESG performance.

**Table 10:** Bootstrap analysis of Type 2 agency costs.

Effect Type	Coefficients	Std. Error	P-Value	95% Confidence Interval
Indirect Effect	0.0014	0.0005	0.005	[0.000406, 0.0023027]
Direct Effect	0.0229	0.0064	0.000	[0.0103658, 0.0354708]
Total Effect	0.0243	0.0064	0.000	[0.0116736, 0.0368717]

### 4.7. Heterogeneity analysis based on enterprise life cycle

The group regression analysis based on enterprise life cycle shows that the impact of DT on ESG performance varies significantly across different life cycle stages. From the ESG-mean results in columns 1–3 of Table 11, there are obvious differences in the extent to which DT influences ESG performance across different enterprise life cycle stages. Specifically, in growth-stage enterprises (column 1), the coefficient of the relationship between DT and ESG performance is 0.0072, but it does not reach statistical significance, indicating that DT in growth-stage enterprises has not yet significantly enhanced their ESG performance. In mature-stage enterprises (column 2), the positive impact of DT on ESG performance is significantly strengthened, with a coefficient reaching 0.0574 and significant at the 5% level, suggesting that mature-stage enterprises can better transform digital technology into sustainable development practices. While in declining-stage enterprises (column 3), DT and ESG performance show a stronger positive correlation, with a coefficient of 0.0582 that is highly significant at the 1% level. This finding suggests that declining-stage enterprises may more actively utilize DT to improve their ESG performance in order to address survival challenges and reshape their corporate image. The ESG median (ESG-med) results in columns 4–6 show a highly consistent pattern with the average value results. The impact coefficient of DT on ESG performance in growth-stage enterprises (column 4) is 0.0088, still not reaching statistical significance; in mature-stage enterprises (column 5), the facilitating effect of DT is significantly enhanced, with a coefficient reaching 0.0754 and highly significant at the 1% level; while in declining-stage enterprises (column 6), the impact coefficient of DT is 0.0696, also highly significant at the 1% level.

**Table 11:** Heterogeneity analysis based on enterprise life cycle.

Variables	(1)	(2)	(3)	(4)	(5)	(6)
	Growth stage	Maturity stage	Decline stage	Growth stage	Maturity stage	Decline stage
	ESG_mean	ESG_mean	ESG_mean	ESG_med	ESG_med	ESG_med
DT	0.0072	0.0574**	0.0582***	0.0088	0.0754***	0.0696***
	(0.010)	(0.026)	(0.021)	(0.010)	(0.028)	(0.023)
Size	0.2300***	0.2208***	0.2111***	0.2390***	0.2346***	0.2016***
	(0.023)	(0.048)	(0.051)	(0.025)	(0.051)	(0.056)
Lev	−0.7598***	−0.5710***	−0.9534***	−0.7525***	−0.4985**	−0.9213***
	(0.081)	(0.203)	(0.183)	(0.087)	(0.220)	(0.196)
ROA	0.7329***	0.3345	0.3540	0.7788***	0.5357	0.4236
	(0.181)	(0.449)	(0.292)	(0.194)	(0.501)	(0.319)
Cashflow	−0.5012***	−0.2109	−0.1142	−0.5909***	−0.1346	−0.1270
	(0.157)	(0.448)	(0.268)	(0.167)	(0.505)	(0.286)
Indep	1.0160***	−0.2029	1.2163***	1.0576***	0.0908	1.1620**
	(0.201)	(0.557)	(0.429)	(0.211)	(0.583)	(0.461)
INST	−0.1599**	−0.5395**	0.3743*	−0.1741**	−0.6107**	0.4746**
	(0.078)	(0.225)	(0.217)	(0.083)	(0.242)	(0.239)
Top1	0.4107**	0.8088*	0.0983	0.3454**	0.6706	0.0445
	(0.168)	(0.454)	(0.411)	(0.176)	(0.466)	(0.456)
Balance	0.0421	0.0164	−0.0481	0.0405	0.0226	−0.0422
	(0.032)	(0.079)	(0.076)	(0.034)	(0.085)	(0.084)
FSTS	0.0210	−0.1437	−0.4798***	0.0378	−0.1488	−0.4888***
	(0.075)	(0.201)	(0.165)	(0.080)	(0.222)	(0.178)
RD	1.4291***	−1.2757	1.2021*	1.2941***	−1.3891	0.9831
	(0.374)	(1.154)	(0.682)	(0.402)	(1.166)	(0.734)
Constant	−1.1701**	−0.5839	−0.9187	−1.3534**	−0.9820	−0.7355
	(0.503)	(1.071)	(1.123)	(0.537)	(1.152)	(1.235)
Observations	16,381	2,584	4,023	16,381	2,584	4,023
Adjusted R-squared	0.496	0.494	0.541	0.433	0.429	0.487

Following the research methodology of Jiang and Bai [70], we conducted rigorous statistical tests on the coefficient differences between different groups (Table 12). Although the grouped regression results show coefficient differences in the impact of DT on ESG performance, this is not sufficient to confirm statistical significance. Therefore, we employed Fisher’s test based on regression coefficient difference testing commands, finding significant differences in DT coefficients between growth-stage enterprises and mature-stage enterprises (−0.05, p-value = 0.066) as well as between growth-stage and declining-stage enterprises (−0.051, p-value = 0.034), while the coefficient difference between mature-stage and declining-stage enterprises (0.001, p-value = 0.484) is not statistically significant. These results strongly confirm that the enterprise life cycle indeed moderates the relationship between DT and ESG, particularly with substantial differences between growth-stage enterprises and enterprises in other stages, possibly reflecting differences in resource allocation and strategic priorities across different life cycle stages.

**Table 12:** Between-group difference tests.

Group Comparison	Coefficient Difference	p-value
Growth vs. Mature	−0.050	0.066
Growth vs. Declining	−0.051	0.034
Mature vs. Declining	0.001	0.484

### 4.8. Further analysis based on ownership nature

Table 13 presents the results grouped by ownership nature, showing that DT’s impact on ESG performance differs significantly between the two types of enterprises. In the state-owned enterprise sample (column 1), the coefficient of DT’s impact on ESG performance is 0.0438 and significant at the 1% level. In contrast, for non-state-owned enterprises (column 2), this coefficient decreases to 0.0173 and is only significant at the 10% level. The between-group difference test further confirms this finding, with a coefficient difference of −0.026 that is significant at the 5% level (p = 0.05), indicating that DT’s positive effect on ESG performance is indeed stronger in state-owned enterprises than in non-state-owned enterprises. This result may stem from two factors. On one hand, state-owned enterprises typically have more abundant resources for digital technology investment and sustainable development practices. On the other hand, as important implementers of government policies, state-owned enterprises have stronger motivations to respond to the dual requirements of national digital strategy and sustainable development goals. When implementing DT, state-owned enterprises are more inclined to integrate it with ESG practices, thereby achieving more significant synergistic effects. These findings suggest that ownership nature, as an important contextual factor, significantly moderates the relationship between DT and ESG performance, further enriching our understanding of DT’s role in promoting corporate sustainable development, and providing a basis for different types of enterprises to formulate differentiated digital transformation and sustainable development strategies.

**Table 13:** Moderation analysis of ownership nature.

Variables	(1)	(2)
	State-owned enterprises	Private enterprises
	ESG_mean	ESG_mean
DT	0.0438***	0.0173*
	(0.015)	(0.010)
Size	0.2778***	0.2370***
	(0.032)	(0.025)
Lev	−0.5669***	−0.7521***
	(0.127)	(0.082)
ROA	0.0214	0.7513***
	(0.262)	(0.154)
Cashflow	−0.1467	−0.3511***
	(0.164)	(0.119)
Indep	1.2423***	0.7915***
	(0.242)	(0.241)
INST	−0.0540	−0.0983
	(0.142)	(0.079)
Top1	−0.4020	0.7063***
	(0.256)	(0.194)
Balance	−0.0881*	0.0673*
	(0.052)	(0.034)
FSTS	0.0275	−0.0378
	(0.109)	(0.076)
RD	0.7985	1.2315***
	(0.645)	(0.333)
Constant	−2.1762***	−1.4177***
	(0.703)	(0.550)
Observations	7,909	15,079
Adjusted R-squared	0.547	0.485
Difference between groups	−0.026(p = 0.05)	

## 5. Conclusions and discussion

### 5.1. Conclusions

This study systematically examines the impact mechanism of DT on ESG performance and its heterogeneous characteristics across different corporate life cycle stages, based on panel data from Chinese A-share listed companies from 2010–2022. The research draws the following main conclusions:

First, DT has a significant positive effect on corporate ESG performance. Through the construction of panel fixed effects models and the use of instrumental variable and system GMM estimation methods, this study finds a robust positive relationship between DT and corporate ESG performance. This finding supports the views of mainstream scholars [10–13], but differs from the inverted U-shaped relationship proposed by Yang and Han [15]. This difference mainly stems from previous studies’ failure to systematically integrate the two key dimensions of “motivation” and “capability,” which led to a one-sided understanding of the relationship between DT and ESG. This study provides a more comprehensive explanation for understanding this complex relationship through a two-dimensional analytical framework of “capability-motivation.”

Second, DT affects corporate ESG performance through the dual pathways of resource effects and governance effects. From a resource dependency theory perspective, DT enhances companies’ ability to implement ESG by alleviating financing constraints. From an agency theory perspective, DT changes management’s intrinsic motivation to advance sustainable development by reducing agency costs. This dual “capability-motivation” pathway mechanism integrates scattered findings in existing literature, thereby providing a more systematic explanation for understanding the complex relationship between DT and ESG.

Third, the promotional effect of DT on corporate ESG performance shows significant differences across different lifecycle stages. This positive impact is more prominent in mature and declining companies, while not significant in growing companies. Different from Li et al. [13]‘s research, this paper reveals the inherent mechanism of this heterogeneity through the “capability-motivation” analytical paradigm. Mature companies possess abundant resource foundations and stable governance structures, enabling them to more effectively transform digital resources into ESG performance. Declining companies, facing survival pressure and legitimacy challenges, are more motivated to utilize DT to enhance ESG performance to reshape their corporate image. Growing companies, due to resource constraints and strategic focus on expansion, have relatively lower attention to ESG, thus weakening the promotional effect of DT.

Fourth, enterprise ownership nature significantly moderates the relationship between DT and ESG, with this promoting effect being more significant in state-owned enterprises. This finding enriches the discussions of Lu et al. [11] and Yang et al. [14] regarding the moderating effect of ownership nature. Compared to private enterprises, state-owned enterprises bear more social responsibilities and policy objectives, and their managers face a more diversified performance evaluation system, prompting them to place greater emphasis on ESG performance. Meanwhile, the financing advantages and policy support of state-owned enterprises also strengthen their efficiency in converting DT into ESG improvements. This conclusion reveals from the dual perspective of “capability-motivation” how ownership nature regulates the impact of DT on ESG by influencing both the resource conditions and intrinsic motivations of enterprises.

### 5.2. Theoretical contributions

This research makes theoretical contributions in the following three main aspects based on existing literature:

Firstly, this research innovatively integrates resource dependence theory and agency theory, constructing a “capability-motivation” analytical framework that systematically explains the inherent logic of how digital transformation promotes corporate ESG performance. Unlike the fragmented characteristics exhibited by previous studies in explaining this relationship [10,12–14], this research validates that DT influences ESG performance through the dual pathways of resource effects (capability dimension) and governance effects (motivation dimension). The resource effect is manifested as DT providing necessary resource guarantees for corporate ESG practices by alleviating financing constraints; the governance effect is reflected in DT enhancing management’s intrinsic motivation to implement ESG by reducing two types of agency costs. This dual theoretical framework not only integrates scattered findings from existing research but also provides a more complete explanatory system for understanding the complex relationship between DT and ESG performance, effectively reconciling the theoretical contradiction between the inverted U-shaped relationship proposed by Yang and Han [15] and the linear positive correlation found by other researchers.

Secondly, this study deepens the life cycle analysis of the DT-ESG relationship through a dual motivation-capability perspective based on Adizes [16] life cycle theory. Although Li et al. [13] had preliminarily discovered that this impact varies across enterprises in different development stages, this research further reveals the theoretical mechanisms behind this heterogeneity. The results show that due to systematic changes in strategic priorities and resource endowments across different life cycle stages, the promoting effect of DT on ESG exhibits significant differences: for growth-stage enterprises focused on market expansion with limited resources, this effect is not significant; for mature enterprises facing slowing growth challenges but possessing abundant resource reserves, this effect is significantly enhanced; for declining enterprises, despite limited resources but a greater need to strengthen social legitimacy, a unique effect pattern emerges. This analysis not only enriches the application of enterprise life cycle theory in the fields of digitalization and sustainable development, but also provides theoretical guidance for enterprises to optimize the synergy between digital and ESG strategies at different development stages.

Finally, by introducing ownership nature as a key contextual variable, this study deepens the understanding of how property rights moderate the relationship between DT and ESG based on the motivation-capability framework. Existing research has neglected the deeper theoretical mechanisms of how ownership nature plays a role in the DT and ESG relationship [11,42]. This study analyzes from the motivation dimension how state-owned enterprise managers’ multiple goal orientations and institutional missions enhance the intrinsic driving force of DT promoting ESG, while from the capability dimension, it dissects how state-owned enterprises’ advantages in aspects such as financing channels strengthen the promoting effect of DT on ESG. This institutional analysis framework based on motivation-capability provides a more systematic and complete theoretical explanation for understanding the moderating role of ownership nature.

### 5.3. Practical contributions

Research findings indicate that digital transformation can effectively enhance corporate ESG performance through multiple mechanisms. This discovery requires business managers to reposition the strategic value of digital technology—not merely as a tool for improving operational efficiency, but as a key lever for driving sustainable development. Based on this understanding, enterprises should construct a comprehensive digital ESG management system, systematically applying advanced digital technologies to core ESG areas such as environmental monitoring and management, responsible supply chain development, and transparent corporate governance, thereby achieving deep integration of technological empowerment and value creation.

Analysis of the heterogeneity across business life cycles reveals that digital-ESG integration strategies should be adjusted according to developmental stages. Growth-stage enterprises, constrained by both limited resources and market expansion pressures, should adopt a “lightweight” digital ESG strategy, prioritizing limited resources toward key areas that strengthen core competitiveness while carefully selecting ESG digitalization projects that require minimal investment yet generate significant impact. These enterprises are particularly suited to establishing modular implementation pathways, flexibly adjusting investment intensity according to business development rhythm, and actively utilizing external shared resources to effectively reduce fixed costs during the initial transformation phase. In contrast, mature enterprises, with abundant resources and stable market positions, should promote deep integration of digitalization and ESG, constructing comprehensive management platforms and evaluation systems, incorporating ESG indicators into core business decision-making processes, and utilizing advanced data analysis technologies to identify ESG risks and opportunities, thereby creating sustainable competitive advantages that are difficult to imitate. For declining enterprises, digital transformation becomes a central pivot for strategic reshaping, focusing on breakthrough innovations in specific ESG areas by developing new business models based on environmental data or creating digital platforms for resource recycling, effectively reshaping corporate image and market recognition, and opening up new growth spaces.

Analysis of the heterogeneity in ownership structures further refines the practical pathways for different types of enterprises. State-owned enterprises should fully leverage their institutional advantages in policy response and resource acquisition, taking the lead in exploring and demonstrating benchmark practices for integrated digital-ESG development. Specifically, state-owned enterprises can establish comprehensive digital ESG governance systems, incorporate relevant indicators into executive assessment frameworks, and promote the deep penetration of digitalization and ESG concepts across all organizational levels, setting standards and examples for the entire industry. In stark contrast, non-state-owned enterprises, with relatively limited resources, should focus more on extracting economic value from digital ESG transformation, establishing strict value-oriented project selection mechanisms, and prioritizing digital ESG projects that directly impact operational efficiency and market competitiveness. These enterprises should adopt a gradual strategy, starting with areas that most directly affect financial performance, while creating market-oriented ESG information disclosure systems, developing innovative business models based on sustainability concepts, and actively building industrial ecosystem alliances to expand market influence while sharing costs.

Through these differentiated implementation pathways based on enterprise characteristics, various types of companies can precisely optimize their integration strategies for digitalization and ESG, achieving strategic synergy between sustainable development and economic growth under their respective constraints, ultimately forming a virtuous cycle where digitalization empowers improved ESG performance. This refined digital-ESG integration approach not only enhances enterprises’ own sustainable development capabilities but also provides strong support for the green and low-carbon transition of the entire economic system.

### 5.4. Research limitations and future directions

Despite making certain contributions at both theoretical and empirical levels, this research still has the following limitations, which also provide directions for future research:

First, at the methodological level, although this research employed approaches such as instrumental variable method and system GMM to mitigate the main endogeneity issues, it still faces potential endogeneity challenges. In terms of measurement error, digital transformation indicators constructed based on annual report text analysis may contain systematic bias, as companies might strategically emphasize or exaggerate their digital transformation achievements in annual reports. Similarly, ESG ratings may be influenced by the subjective standards and methodologies of rating agencies, limiting the measurement precision of the research variables. Regarding sample selection bias, this research only focused on a specific group of A-share listed companies. These companies generally possess relatively abundant resource foundations and well-established information disclosure mechanisms, and are subject to stricter market regulation. Therefore, the external validity of the research findings may be limited, making it difficult to directly generalize to non-listed companies, small and medium-sized enterprises, or companies operating under different institutional environments. Future research could consider adopting quasi-natural experimental designs, diversified digital transformation measurement indicators, or conduct in-depth investigations based on more diverse enterprise samples, thereby further alleviating these endogeneity issues and enhancing the reliability and applicability of research conclusions.

Second, regarding the research sample, the conclusions drawn from the empirical data of China’s A-share listed companies have important reference value for emerging economies like China, but there may be differences in applicability across different economic backgrounds. The mechanisms and pathways of digital transformation promoting ESG performance discovered in this research are particularly applicable to emerging economy contexts that are in a stage of rapid digital transformation development, gradual standardization of ESG practices, and active government policy guidance. However, considering the differences between countries in terms of digital development stages, ESG regulatory maturity, and cultural backgrounds, the research conclusions may need to be adjusted according to local characteristics in cross-national contexts. For example, Western developed countries generally have more mature ESG evaluation systems and stricter environmental regulations, so the association mechanisms between digital transformation and ESG performance might present different characteristics compared to China. In developing countries with lower levels of digital infrastructure development, the conditions for advancing digital transformation and its implementation effectiveness may lead to different impact pathways on ESG. Therefore, future research could adopt cross-national samples to examine the relationship differences between DT and ESG performance under different development stages and institutional environments. This would help validate the applicability conditions of this research’s conclusions across different economies and provide more targeted practical guidance for companies in various countries.

Third, regarding research content, this study primarily focused on the impact of DT on overall ESG performance, but did not deeply analyze the differentiated impact mechanisms on the three dimensions of environment, social, and corporate governance. Future research could further break down ESG indicators to explore whether differences exist in the intensity and pathways of DT’s impact on each dimension. This would help develop a more nuanced understanding of the application value and implementation strategies of digital technology across different areas of sustainable development.

Fourth, regarding theoretical perspective, although this study integrated resource dependence theory and agency theory to build an analytical framework, it did not fully consider other theoretical perspectives that might affect the relationship between DT and ESG, such as institutional pressure, organizational learning, and dynamic capabilities. Future research could further expand the theoretical foundation, construct a more comprehensive analytical framework, and deepen the understanding of multi-level and multi-pathway mechanisms through which DT promotes ESG performance.
